# Higher ascent, trouble breathing: High altitude pulmonary edema (HAPE)

**DOI:** 10.11604/pamj.2018.30.43.15181

**Published:** 2018-05-18

**Authors:** Simant Singh Thapa, Buddha Basnyat

**Affiliations:** 1 Department of Internal Medicine, Saint Vincent Hospital, Worcester, MA, USA; 2 Nepal International Clinic, Kathmandu, Nepal, Oxford University Clinical Research Unit-Nepal, Center for Tropical Medicine and Global Health, University of Oxford, UK

**Keywords:** Hypoxia, travelers´, high altitude pulmonary edema, USA

## Image in medicine

A 40 year old man started his trekking 4 days ago from Lukla (2800m) in the Everest region of Nepal. He reached Tengboche (3860 m) on third day where he was short of breath with moderate exertion and was easily fatigued. He ascended further ignoring his symptoms and reached Dingboche (4410m) on fourth day. He was extremely tired, short of breath at rest and appeared ill so helicopter evacuation was done from Dingboche to Kathmandu (1300m) and he was brought to our clinic. On initial evaluation, his vital signs were temperature 97.8 F, blood pressure 124/66 mmHg, pulse rate 89/min, respiratory rate of 26 breaths/min and saturation (SpO2) of 80% on room air at rest. Auscultation of the lungs revealed bilateral crackles. Finger to nose test and tandem gait was normal. The chest radiograph showed patchy infiltrates bilaterally. He was diagnosed as the case of HAPE. He was treated with bed rest and supplemental oxygen. On evaluation the next day, his pulmonary crackles had resolved and he was no more short of breath. His ambulatory SpO2 was 96% on room air. His dramatic clinical improvement following descent strongly supported our diagnosis. HAPE is a non-cardiogenic form of pulmonary edema. Its notable feature in contrast with other causes of pulmonary edema is fast reversibility following timely descent with or without oxygen.

**Figure 1:**
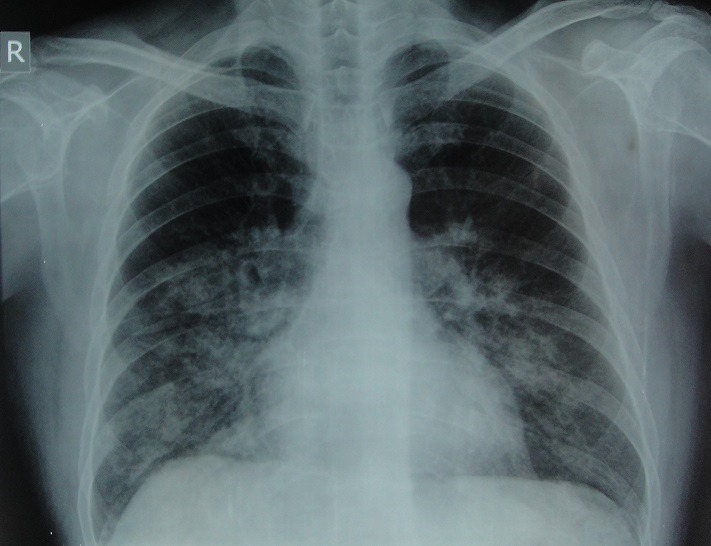
Chest X-ray showing bilateral patchy nodular infiltrate, classically seen in HAPE

